# Utilisation of obstetric sonography at a peri-urban health centre in Uganda

**Published:** 2010-12-28

**Authors:** Mubuuke Aloysius Gonzaga, Elsie Kiguli-Malwadde, Francis Businge, Rosemary Kusaba Byanyima

**Affiliations:** 1 Radiology department, School of Medicine, College of Health Sciences, Makerere University, Uganda; 2 Radiology department, Mulago National Referral Hospital, Uganda

**Keywords:** Obstetric, sonography, Uganda, ultrasound

## Abstract

**Background:**

Maternal mortality is related to obstetric complications in pregnancy some of which could be revealed by obstetric sonography. Obstetric sonography has become part of routine antenatal care in both urban and rural settings. The objective of the study was to assess the utilization of obstetric sonography in a rural hospital of Uganda, including the frequency and appropriateness of its usage as well as determine whether there was any relation between number of obstetric scans, patient management and obstetric outcomes.

**Methods:**

It was a retrospective study in which review of all obstetric charts and obstetric scan requisition forms for all deliveries in Ndejje Health Centre (Uganda) was done.

**Results:**

During the study period, there were 105 singleton deliveries, and these mothers underwent a total of 232 obstetric scans. More than half (53.4%) of the scans were classified as inappropriate. There were no significant differences in the number of scans between low- and high-risk pregnancies or between uncomplicated deliveries and those in which induction or instrumental or operative delivery occurred, nor was there any relation between number of scans and obstetric outcome.

**Conclusion:**

Obstetric sonography has become popular in rural health settings as part of antenatal care. However, it was over-used in the health centre. This overuse was not associated with any identifiable effect on obstetric outcome. Therefore, more appropriate use of obstetric sonography, in accordance with evidence-based guidelines, is recommended.

## Background

The high rate of maternal mortality world-wide is an increasing concern. Direct causes of maternal deaths are related to obstetric complications of pregnancy, labour and childbirth some of which can be detected by ultrasonography [1]. Access to medical services like obstetric sonography should be an important component of healthy policy in developing countries [2]. The higher emphasis on curative than preventive health care has also been noted to be one of the causes of increase in maternal mortality [3,4]. Obstetric ultrasonography, unarguably, is an important aspect of antenatal care, which care forms the foundation of all health care for pregnant women. Observational studies support that antenatal care improves pregnancy outcome [5-8]. Good quality antenatal care can reduce many risks of death, sickness, and disability for both mothers and infants. A study has also shown higher antenatal coverage in urban than in rural areas [8].

Identification of high-risk pregnancies early in prenatal care is widely used to anticipate and, when possible, prevent maternal and fetal morbidity [9,10]. Most risk assessment systems seek to identify demographic features, the aspects of the patient's obstetric history and events in the current pregnancy that put either the mother or the fetus, or both, at risk [11,12]. These risk assessment systems form the basis for risk-scoring on provincial prenatal forms [13,14]. In addition, certain screening procedures, including prenatal ultrasonography, are suggested to aid physicians in following the pregnancy to term [15-17]. For example in current Canadian practice, prenatal sonography is used for 2 different types of screening. In the first type, a single ultrasound examination is done in the second trimester for the purposes of estimating gestational age and detecting major malformations or multiple fetuses. In the second type, 2 serial examinations are performed, one each in the second and third trimesters, to screen for intrauterine growth retardation (IUGR).

The Canadian Task Force on the Periodic Health Examination [17] using meta-analysis of the published trials determined that there is fair evidence to support a single screening prenatal ultrasound examination in normal pregnancy without a specific clinical indication (Class B indication). While giving this tentative support for routine screening, the task force recognized that such a guideline might lead to increased utilization of prenantal sonography [17]. Greater controversy surrounds the use of routine serial ultrasonography to screen for intra-uterine growth retardation. Given that the evidence demonstrating improved outcomes with serial ultrasonography is poor, [18,19] the task force recommendation is a class C recommendation, i.e., there is no evidence to include or not include such screening in prenatal care in low-risk pregnancy [17]. The choice of whether to perform serial ultrasonography has so far been left to the attending clinicians. It appears that even the most experienced obstetricians are moving toward greater use of diagnostic tests to support their management decisions [20,21]. One would expect, then, that in an isolated rural location, there would be even more conservative management of potentially high-risk pregnancies. At the same time, fiscal restraints dictate that health care resources be selectively allocated and that ineffective techniques and practices be identified and eliminated. Although each rural site is different and each is bound by the availability of diagnostic and support services, an attempt is needed to determine what resources are best used to manage both high- and low-risk obstetric patients in rural settings, so that both the patient and the health care system are better served.

In Uganda, obstetric sonography has been very popular amongst women [22]. However, there is little current data on the utilization of obstetric sonography in lower health facilities. Additionally, ultrasound services have been decentralized in Uganda to lower health facilities in order to bring services nearer to people [23]. However, there is a dearth of literature documenting the utilization of such services. The purpose of this study therefore was to determine the frequency of obstetric sonography in a peri-urban health centre in Uganda; to assess the appropriateness of the scans; to assess the relation between prenatal sonography and the management of the pregnancy as well as that between prenatal sonography and maternal and neonatal outcomes. Throughout this paper, the words ultrasound, scan, sonography will be used interchangeably.

## Methods

**Study setting**

The study was conducted at Ndejje Health Centre in Uganda. Ndejje health centre is at level IV in the decentralized health care structure in Uganda serving a population of about 100,000 people. It is a peri-urban health centre located in Wakiso district just at the outskirts of Uganda's capital, Kampala. Health centre IV facilities in Uganda offer ultrasound services.

**Study design**

It was a retrospective observational study from January 1st to December 31st, 2008. All requests for obstetric sonography and all delivery reports from the health centre were reviewed during that period. From the request forms, reasons for each obstetric scan were recorded, and from the subsequent reports, the calculated gestational age, any abnormalities of the fetus or placenta that were reported, and suggestions made were recorded. Data were also abstracted from the charts of both the mother and the newborn including the hospital prenatal and live-birth records. This data included maternal age, parity, gestational age at delivery, use of sonography, results of gestational diabetes screening, presence or absence of hypertension, other risk factors, whether labour was induced or augmented, type of delivery, use of episiotomy, maternal complications at delivery and any fetal complications. In addition, the number of referrals was documented. In this study population, the factors leading to an initial designation of high risk included prior cesarean section, previous complicated obstetric history, pre-existing medical problems, age of primigravida less than 16 years or greater than 35 years and obesity.

All the obstetric scans were classified by the investigators as “appropriate” or “inappropriate”. This classification was purely based on a previous study done in this area [25], but not just formulated by the authors.

Appropriate

1) Any scan ordered for specific medical reasons, i.e. bleeding, abdominal pain, size–date discrepancies, results of biophysical profile or inability to determine fetal position clinically, regardless of gestational age, 2) Any examination ordered between 10 and 24 weeks gestational age for the purposes of dating and screening for congenital abnormalities.

Inappropriate

1) Any examination ordered for dating before 10 weeks or between 20 and 28 weeks gestational age. Before 10 weeks little information can be gained about fetal morphologic features, and after 20 weeks dates based on menstrual history are generally as accurate for assessment of gestational age18. We also recorded whether additional sonographic examinations were required because of this suboptimal examination, 2) Any examination ordered beyond 20 weeks gestational age for the indication of routine monitoring of growth (with no clinical evidence of IUGR). Such imaging was classified as inappropriate because there is no concrete evidence to support this indication; 3) Any examination performed as a result of a suggestion by the radiologist/sonographer to repeat the scan because of the technical inability to conclusively demonstrate placental position.

**Data analysis**

Data was analysed with Epi-Info statistical package. Descriptive statistics were compiled for ultrasound use, patient demographics and other medical information about the management of the pregnancies. One-way analysis of variance was used to test differences between means. A pvalue of 0.05 was identified as the level of statistical significance.

**Ethical issues**

Permission to carry out this study was granted by the Radiology department Education and Research Committee as well as the In-Charge Clinician of Ndejje Health Centre. All patient information accessed was kept confidential and secure in a locked place, and only the researchers had access to it. No patient name or any form of identification was retrieved from the records to include in the data. Raw data was entered into a private computer accessed by only the researchers and was protected by a password.

## Results

During the study period, 105 women delivered babies beyond 28 weeks gestational age at Ndejje health centre. The overall mean age was 27 years, that for primigravid women 25.2 (range 17 to 38) years and that for multigravid women 30.7 (range 20 to 43) years. Table 1 shows data about the study population. Eighty five women (80.9%) were considered at low risk at the onset of pregnancy, and 20 (19.1%) were identified as being at a high potential risk, on the basis of factors identified in the records. One-way analysis of variance showed significantly fewer obstetric scans for women with post-term delivery (p<0.05).

**Ultrasound evaluation results**

There was no Radiologist at the health centre. A total of 232 scans were requested for the 105 women who delivered at the hospital, an average of 2.2 scans per delivery. All the women in this study had at least one obstetric scan, 90 (85.7%) underwent at least two, and 50 (47.6%) had three or more, which accounts for the additional scans done. Of the 232 examinations, 124 (53.4%) were classified as inappropriate (Table 2). Of these, 70 (56.5%) were ordered for serial monitoring of growth, 40 (32.3%) were inappropriately timed, and 14 (11.2%) were ordered because of a technical problem (Table 3). One-way analysis of variance revealed no significant differences in the number of scans between the various categories for gravidity, risk level, type of labour, type of delivery, maternal postpartum complications and neonatal complications (for all comparisons, p >0.05). However, for the length of gestation, patients with post-term delivery underwent significantly fewer scans (p <0.05).

## Discussion

There has been limited literature assessing the utilization of obstetric sonography in lower health facilities in Uganda. The available literature has mainly focused on large health settings [22]. In this study, it was found out that obstetric sonography was equally popular in smaller health facilities where it is available. The possible explanation for this popularity could be due to the emphasis put on obstetric sonography as part of routine antenatal care even by the health workers themselves. At the same time, sonography is now widely available in many health facilities in Uganda. Such findings have also been reported by Thompson et al [25]. The lack of a Radiologist at the health centre is the reason why a radiographer carried out all the scans. There are very few Radiologists in Uganda mostly found in large urban centres and as such, lower level health facilities lack this specialized cadre of staff.

Regarding the appropriateness of the first obstetric scan, many of the scans were requested at inappropriate times. Even with the time limit allowed in this study for the first scan at 10 to 20 weeks gestational age, compared with the usual recommendation of 12 to 18 weeks [25], it was found that many of the scans had been done at a suboptimal time. This is an issue that could possibly be addressed through clinician education since it is the clinicians who request for the scans. The situation got worse with the second and subsequent scans, majority of which were judged as unnecessary or inappropriate. The easy availability of obstetric sonography probably has contributed to clinicians requesting for it even when it is not necessary. Therefore, there is need probably to sensitize clinicians on the appropriate times when obstetric scan are most useful to them and the pregnant women.

**Relation between obstetric sonography and maternal and neonatal outcomes**

If obstetric scans are being performed in the hope of detecting abnormalities in either the baby or the mother, it might reasonably be expected that more such scans would be performed in cases in which maternal or neonatal complications subsequently developed. However, no such evidence was found to support this hypothesis: the number of obstetric scans requested were not significantly different between mothers who remained at low risk and experienced no complications and women who underwent cesarean section, who had induced or augmented labour, who had originally been classified by the prenatal risk-scoring system as being at high risk, who experienced complications during or after labour or whose babies experienced complications. These findings agree with those reported in earlier studies [18,19,25]. In fact, in this study fewer scans were performed for women who delivered post-term, a group for whom biophysical profiles are commonly requested. The reason for this difference is unclear. The results of biophysical profiles are a recognized indication for late trimester scans [28] although no profiles were requested in the records reviewed. A possible explanation for the difference could be due to lack of technical expertise. There was no radiologist on site and the radiographer who performed the scans was probably not very competent.

**Possible reasons for the many obstetric scans**

Several factors may have contributed to the high rates of multiple obstetric scans. The first would seem to be the apparent high rate of reports that advised for repeat of the scan later on to evaluate the fetus or rule out maternal abnormalities; so women had to come back for follow-up scans. The emphasis by clinicians for obstetric sonography as a routine checkup is another possible explanation for the many scans requested. This brings about the issue of overuse of obstetric sonography even when it is not necessary. Many women ask the health workers to request a scan for them, while at the same time many health workers routinely include sonography as part of antenatal care even when it is not necessary. This overuse of obstetric sonography was reported by Mubuuke et al [22] in an earlier study. Gammeltoft and Nguyen [26] as well as Tautz et al [27] have almost reported similar reasons for the overuse of obstetric sonography in other parts of the world. However, it should be advanced that such routine imaging adds significant costs to the women and may not yield any more valuable information as thought.

In addition, the scans were performed by the radiographer and could not be reviewed immediately by a radiologist where need arose, a practice more common in larger urban centers. Because the information obtained from scan depends both on fetal gestational age and on the competency of the operator, [15] this situation may have led to more repeat scans where the radiographer was not sure and needed to follow up. We wonder whether the repeat scans could have been avoided if the patients had undergone imaging at 14 to 18 weeks gestational age at a larger centre with more skilled sonographers and radiologists on site.

Another factor was the use of obstetric scans at inappropriate times. Many dating scans were performed too early to yield optimal information on gestational age or fetal and placental abnormalities; furthermore, the screening obstetric scan was performed somewhat late (between 20 and 26 weeks) in many cases. Limiting such “screening ultrasonography” to the recommended period of 14 to 18 weeks after the last menstrual period would certainly yield more accurate information and would remove the need for many more follow-up scans.

**Limitations of the study**

This was a retrospective observational study. As such, the data were amenable to weak inferences as it was assumed that all of the relevant information needed had been recorded in the charts of mothers and newborns and on the ultrasound request forms. Many of the ultrasound request forms lacked some vital data like menstrual history. For example menstrual history and age as determined by sonography were not compared, although this might have clarified the indications for some of the “inappropriately” indicated scans. In addition, only one health centre was selected for the study. It is quite hard to say with certainty that the group of women at this one health centre was representative of the general maternity population in all other lower level health centres.

## Conclusion

The use of obstetric sonography has been advocated for even in rural settings. There is therefore need to train more professionals to provide this service in rural areas. However from this study, too many obstetric scans inappropriately requested for with no identifiable effect on outcomes or the management of labour. There is therefore need for more judicious use of obstetric sonography only when it is appropriate. This study has identified issues for future research and has perhaps shown where better clinician education might lead to a reduction in the misuse of obstetric sonography that subsequently reduces the costs for both patients and the health care system.

## Figures and Tables

**Table 1:** Characteristics of the 105 singleton deliveries that occurred at Ndejje Health Centre in relation to frequency of obstetric sonography

**Characteristic**	**Number of cases n (%)**	**Number of obstetric scans mean (and SD)**
**Gravidity**		
Primigravida	57 (54.3)	2.21 (0.71)
Multigravida	48 (45.7)	2.09 (0.90)
**Risk**		
Low	85 (80.9)	2.29 (0.89)
High	20 (19.1)	2.14 (0.70)
**Length of gestation**		
Pre-term (<37 weeks)	3 (2.9)	2.03 (1.01)
Full term (37-41 weeks)	97 (92.4)	2.24 (0.76)
Post-term (>41 weeks	5 (4.8)	1.19 (0.99)
**Type of labour**		
Spontaneous	72 (68.6)	2.12 (0.80)
Augmented	26 (24.8)	2.20 (0.80)
Induced	7 (6.7)	2.02 (0.60)
**Type of delivery**		
Vaginal	60 (57.1)	2.18 (0.87)
Forceps or vacuum	10 (9.5)	2.10 (0.60)
Cesarean section	35 (33.3)	2.15 (0.80)
**Postpartum complications**		
Present	12 (11.4)	2.20 (0.68)
Absent	93 (88.6)	2.15 (0.84)
**Neonatal complications**		
Present	10 (9.5)	1.70 (0.48)
Absent	95 (90.5)	2.22 (0.90)

**Table 2:** Appropriateness of first, second and subsequent Obstetric scans in each pregnancy at Ndejje Health Centre

**Obstetric scan**	**Appropriate**	**Inappropriate**	**Total**
First	78	25	103
Second	11	80	91
Subsequent	19	19	38
**Total**	**108**	**124**	**232**

**Table 3:** Reasons for designating obstetric scans as inappropriate

**Obstetric scan**	**Sub-optimal timing**	**Scan ordered for technical reasons**	**Scan ordered for “serial” monitoring**	**Total**
First	27	0	5	32
Second	13	11	50	74
Subsequent	0	3	15	18
**Total**	**40**	**14**	**70**	**124**
